# Can Serotonin 7 Receptors Be a Treatment Target for Noncentral Diseases?

**DOI:** 10.5152/eurasianjmed.2023.23303

**Published:** 2023-12-01

**Authors:** Aslı Özbek Bilgin

**Affiliations:** Department of Pharmacology, Erzincan Binali Yıldırım University, Erzincan, Turkiye

**Keywords:** Serotonin, 5-HT, 5-HT7, receptors

## Abstract

The neurotransmitter serotonin (5-HT) is involved in several key processes in the central nervous system. But the great majority of serotonin is produced by intestinal enterochromaffin cells of the gastrointestinal (GI) tract and circulating blood platelets, which work independently of the central nervous system. The mediating pathway through which serotonin transmits its effects is the 5-HT receptor (5-HTR) superfamily, which consists of at least 14 members with extensive characterizations. Having been discovered as the final member of the 5-HTR family, 5-HT7 receptors have specific roles in the neurological, GI, circulatory, and immune systems. Due to their extensive distribution, these receptors’ stimulation and repression are of importance during the therapy process, even if their exact mechanism of action in disease has not been fully understood. This review establishes the functions of 5-HT7Rs in systems and discusses how these receptors may be used therapeutically to treat peripheral diseases.

## Introduction

Serotonin (5-HT) has been defined as the “happiness hormone” and has gained attention in the central nervous system as a therapeutic target for mood disorders and neuropsychiatric illnesses. But serotonin’s impact goes far beyond its correlation with specific brain activities. An increasing amount of research on the relationship between 5-HT and the gut microbiota has emerged recently, indicating that 5-HT may be crucial along the brain–gut–microbiome axis.^1^ Enterochromaffin (EC) cells in the gastrointestinal (GI) tract in humans create about 90% of 5-HT from dietary tryptophan, which is involved in central (5%) and peripheral (95% in the GI tract for digestive processes) functions.^2,3^ Serotonin has a wide range of receptors in various tissues, like some compounds,^4-8^ which makes it a highly versatile neurotransmitter and also an autocoid. The serotonin receptor subtypes, the covalent binding of serotonin to various effector proteins, and the serotonin transporter are the mechanisms by which serotonin carries out its tasks.^9^ To date, 7 distinct families and 14 various subtypes of 5-HT receptors (5-HTR) have been determined.^10^ Among these receptor types, 5-HT7 receptors (5-HT7R) are the newest subfamily and are known to be expressed highly in brain areas that are essential to function and peripheral tissues.^11,12^

Primary sites of 5-HT7R expression in the central nervous system include the thalamus, hippocampus, prefrontal cortex, hypothalamus, amygdala, basal ganglia, and dorsal raphe nucleus. For instance, 5-HT7R regulates cognition, processing of memories, and perception of emotion, as shown by its expression in the limbic areas.^13-15^ Additionally, 5-HT7R expression has been noted in the stomach, arteries’ smooth muscle cells, pancreas, liver, kidney, and GI tract.^13,16^ Furthermore, these results imply that 5-HT7R regulation can be a valuable therapeutic target for disorders affecting peripheral tissues as well as the central nervous system.

According to preclinical research, 5-HT7R has been linked to numerous physiological and pathological processes, such as pain and migraine, irritable bowel syndrome, sepsis, hepatotoxicity, carcinogenesis, immune cells, blood pressure, thermoregulation, and circadian rhythms.^17-24^

This contribution focuses on conducting a systematic analysis of reports on the function of the 5-HT7Rs and the curative effects of agonists/antagonists in peripheral pathophysiologic circumstances.

## Distribution of 5-HT7 Receptors and Intracellular Signaling Pathways

The International Union of Basic and Clinical Pharmacology (IUPHAR) has named and classified 5-HTR as one of the other receptors within the framework of certain rules. With the latest updates, the information about G-protein-coupled 5-HT7 receptors was determined by Andrade et al.^25^

**Figure 1.**
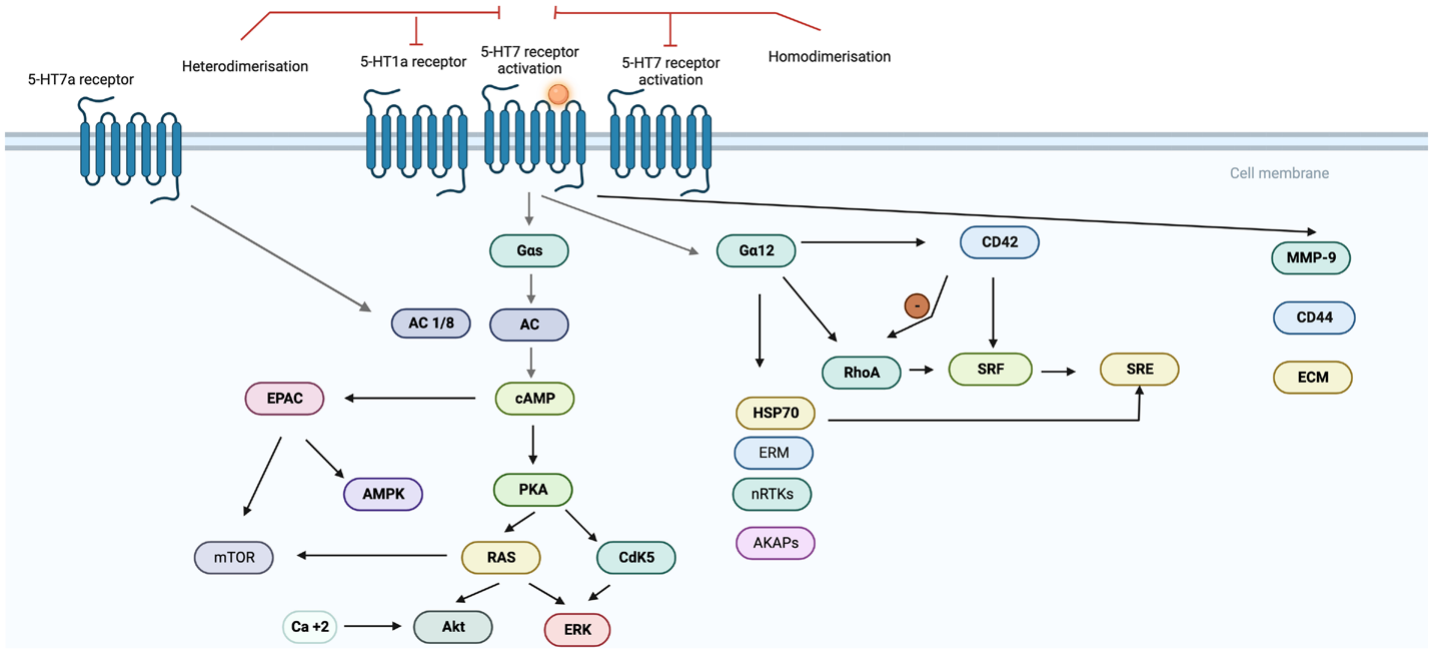
Signaling pathways regulated by the 5-Ht7 receptor. AC, adenylyl cyclase; cAMP, cyclic adenosine monophosphate; PKA, protein kinase A; ERK, extracellular signal-regulated kinases; Akt, protein kinase B, Hsp90, heat shot shock protein 90; ERM, proteins of the ezrin–radixin–moesin family; GEF, guanine nucleotide exchange factor (represented by the leukemia-associated RhoGEF LARG and p115Rho); nRTKs, non-receptor tyrosine kinases; AKAPs, A-kinase anchoring proteins; SRF, serum response factor; SRE, serum response element; AMPK, adenosine monophosphate-dependent protein kinase; Cdc42, cell division cycle protein 42; Cdk5, cyclin-dependent kinase 5; EPAC, exchange protein directly activated by cAMP; mTOR, mammalian target of rapamycin; RhoA, Ras homolog gene family member A; CD44, hyaluronic acid receptor; MMP-9, metalloproteinase 9; ECM, extracellular matrix.

The gene of 5-HT7R was cloned in rats,^26-28^, mice,^29^ human,^30^ and guinea pig^31^ by molecular cloning analyses performed in different laboratories in 1993. It is found in humans and codes for a 445-amino acid protein having an open reading frame of 1335 base pairs.^30^ 5-HT7a, 5-HT7b, 5-HT7c, and 5-HT7d are 4 distinct splice variants; nonetheless, all of them are functionally active. The 5-HT7a, 5-HT7b, and 5-HT7d receptors were identified in humans. Despite having varying intracellular carboxyl-terminal (C-terminal) tails, the splice variants exhibit comparable constitutive activity and effects when an inverse agonist binds to them.^32^ An exception to this is the human 5-HT7d receptor, which possesses a distinct internalization pattern that can impact receptor-mediated signaling.^33^ Regarding this, the 5-HT7d receptor was found to be permanently integrated in the lack of an agonist, indicating that, unlike the 5-HT7a and 5-HT7b receptors, it may have a pattern interacting with machinery of cellular transport in its longest known human 5-HT7 receptor isoform’s carboxyl-terminal tail.^34^ Through the activation of Gαs, Gα12, and metalloproteinase 9, all 4 of these 5-HT7R splicing variants are linked to G protein and impact 3 intracellular signaling pathways^26,35,36^ (Figure 1).

### Gαs Signaling Pathway

Adenylate cyclase activity, ligand-binding affinity, and localization of 5-HT7 splicing variants have not been found to differ significantly. Conversely, the 5-HT7a isoform specifically triggers cyclic adenosine monophosphate (cAMP)-dependent signaling by activating 1 and 8 types of adenylyl cyclase via Gs-independent and Ca^2+^/calmodulin-dependent signaling.^37^

When the 5-HT7R is activated, a series of events take place, including the production of cAMP through adenylyl cyclase activation, which subsequently interacts with a variety of intracellular targets. Increasing cAMP triggers the exchange protein directly activated by cAMP (EPAC) and protein kinase A (PKA) to signal.^35^ By phosphorylating target proteins, each of these signaling pathways influences different signal transductions and causes the signal to spread to ensuing metabolic activities. Activation of PKA then triggers Ras and cyclin-dependent kinase 5, which activates serine/threonine extracellular signal-regulated kinases (ERK) signaling^38,39^ via inhibitory PKA and stimulatory EPAC, the other pathway manages adenosine monophosphate-dependent protein kinase (AMPK). Additionally, the activation of mTOR is facilitated by 5-HT7-induced Ras and EPAC signaling, whereas the mammalian target of rapamycin mTOR signaling is suppressed by activated AMPK.^40^ Erk signaling is also indirectly improved by EPAC activation.^39^ Significantly, increases in intracellular Ca^2+^ and cAMP are necessary for the 5-HT7R-mediated activation of protein kinase B (Akt), whereas Ca^2+^ inhibits the activation of ERK. It is known that Gαs-protein activation via the 5-HT7 receptor may be responsible for effector molecule activation that controls cytoskeleton development and cellular motility.^34^

## Gα12 Signaling Pathway

The transcription factor called serum response factor binds to the serum response element (SRE), is activated in a Rho-dependent manner upon 5 HT7R-mediated stimulation of Gα12-protein. Notably, despite the presence of a PKA inhibitor or pertussis toxin, stimulation of the 5-HT7R resulted in a dose-dependent increase in SRE-driven gene expression, indicating receptor-mediated SRE activation in a PKA-independent manner.^35^ A Rho-independent mechanism of Gα12-mediated SRE activation via Hsp90 has also been elucidated.^41^ 5-HT7/Gα12 has been shown to activate cell division cycle protein 42 (Cdc42) as well as Ras homolog gene family member A (RhoA),^35^ indicating a hierarchical cascade between the RhoA and Cdc42 pathways wherein Cdc42 downregulates RhoA activity.^35,42^

### Other Pathways

A different pathway connected to 5-HT7 stimulates metalloproteinase-9, which cleaves the hyaluronic acid receptor’s extracellular domain and causes the receptor to separate from the extracellular matrix.^36^ Dynamic palmitoylation of the 5-HT7R (bound to both Gαs and Gα12 proteins) might be a mechanism that regulates selectively Gαs- or Gα12-mediated signaling.^34^ Since homo- and heterodimerization may serve as further regulatory mechanisms for GPCR-mediated signaling, it has also been shown that 5-HT7R may form homo-oligomers in the recombinant system and he–terodimers with 5-HT1AR *in vivo* and *in vitro*.^43^

## Preclinical Findings About the Therapeutic Potential of Peripheral 5-HT7R Modulation

As many molecules are reported as agonists, inverse agonists, and antagonists of 5-HT7R,the use of these and a knockout mouse has helped to define the functions of the 5-HT7Rs.^44^

### Immune and Inflammatory Effects

Although the underlying mechanisms remain unclear, it is becoming increasingly evident that serotonin, a peripheral neurotransmitter, plays a significant role in immunity as well as in inflammatory and immunomodulatory illnesses. It is theoretically possible for immunological responses to be influenced by central, neuronal serotonin or by peripheral, mostly platelet-derived serotonin.^9^ As is known, many immune cells synthesize, transport, store, and/or respond to 5-HT and express its receptors.^45^

5-HT7Rs are considered to be found in immunological cells in lymphatic tissues dispersed throughout the GI system, including monocytes, lymphocytes, and dendritic cells (DCs), in addition to gut-associated neurons. These cells may be important players in the signaling of inflammation.^34,45,46^ Neutrophils have 5HT7Rs as well, yet it is unknown how 5-HT7R signaling and regulation affect neutrophil activity overall. Evidence currently available shows that the 5-HT7R has no function in neutrophil recruitment, despite the fact that serotonin’s action on other receptors can modulate neutrophil migration.^47^ Additionally, immunological tissues such as the thymus and spleen, bone marrow-derived mononuclear cells, and peripheral blood DCs have 5-HT7Rs.^48^

When the 5-HT7R is activated in mature DCs, interleukin (IL)-1β and IL-8 are released, but IL-12 and tumor necrosis factor production is decreased.^49^ 5-HT7 receptors are expressed by native splenic T cells; ex vivo exposure to 5-HT causes a 5-HT7R-dependent activation of ERK 1/2, an increase in the rate of proliferation, and an increase in the expression of CD25; the 5-HT7R antagonist SB 269970 reverses this response.^46^ All of this indicates that T-cell reactions to inflammatory stimuli involve 5-HT7 receptors.

Experimental studies on dextran sodium sulfate (DSS)-induced skin fibrosis and colitis in mice have shown that by chemically or genetically disrupting 5-HT7 receptor signaling, macrophage infiltration and collagen deposition are inhibited.^50^ Pro-fibrotic gene signature is promoted by 5-HT7R signaling in a manner that is dependent on PKA and 5-HT7.^50^

Albayrak et al^51^ reported that in response to inflammatory stimuli, expression of 5-HT7Rs in rat paw tissue was observed. Additionally, it is shown that the 5-HT7R agonist AS-19 has an anti-inflammatory effect comparable to that of the common anti-inflammatory medication indomethacin and that co-administration of the antagonist pharmacologically reversed this effect. The anti-inflammatory effect of agonist treatment on paw tissue was associated with a decline in the serum cytokine response to carrageenan, a decline in COX-1 and COX-2 activity, and an increase in the antioxidant system despite a decrease in oxidative stress. They suggested that, for the purpose of reducing inflammation and inflammatory disorders, 5-HT7R can represent a viable novel therapeutic target.^51^

### Effects on the Gastrointestinal Tract

Since 5-HT appears to be the cause of 5-HT-induced smooth muscle relaxation in the ileum of guinea pigs, the stomach of dogs, and the colon of humans, intestinal motility has been suggested to be influenced by 5-HT7 receptors. Although this is still up for debate, 5-HT7 might also play a part in peristalsis.^52^

5-HT7 receptors appear to mediate mucosal 5-HT’s pro-inflammatory actions, indicating that this receptor may be involved in gut illnesses like inflammatory bowel disease (IBD).^53^ It is reported that targeting the 5-HT7 receptor by SB-269970, an antagonist, reduced DSS-induced colitis in acute and long-term conditions. Based on this, the 5-HT7R is suggested to be essential for controlling immune responses and mucosal inflammation. Also targeting the 5-HT7R on DCs could be a useful therapeutic method to reduce mucosal inflammation and treat GI inflammatory conditions such as IBD.^54^ Guseva et al,^55^ however, in contrast, presented that the DSS model exhibits an elevated inflammatory response when 5-HT7 is disrupted genetically or by the drug SB-269970.

Also, more recent studies revealed that 5-HT7 might be involved in the physiopathology of Crohn’s disease (CD) and ulcerative colitis (UC), the main types of IBD. In inflammatory sections of CD patients and biopsies of the colon from UC patients in remission, the elevation of 5-HT7 expression in DC was shown.^55,56^

Another study by Calik et al^57^ hypothesized that 5-HT7 receptors have critical roles in the occurrence of gastric ulcers and that 5-HT7Rs might genuinely have a role in the protective mechanism of stomach cells. It has also been stated that it is possible to prevent stomach ulcers by activating 5-HT7 receptors as a potent antiulcer therapy, but its inhibition increases the risk of gastric ulcers caused by indomethacin, so caution should be exercised when using 5-HT7 antagonists in both clinical and preclinical studies.^57^

### Effects on Lungs

Numerous studies have noted that 5-HT7R may be implicated in several respiratory system functions. While 5-HT7R may have a role in regulating the motor output of the phrenic nerve, it has also been suggested that 5-HT7R has an inhibitory effect on the hypoxic respiratory response. These findings imply that 5-HT7R might be essential for respiratory flexibility in hypoxic environments.^58^

It’s interesting to note that 5-HT7R may control inflammatory reactions in lung tissues and may be a destination for the management of lung sepsis. Serotonin 7 receptor activation reduced lung tissue damage and inflammation in 2 different models of sepsis. In short, 5-HT7R agonist AS-19 improved life duration and reduced lung oxidative stress, the response of serum cytokines, and lung injury caused by sepsis treated with cecal ligation and puncture (CLP) in rats.^24^ Lipoprotein shock (LPS)-induced sepsis in rats was similar to CLP-induced sepsis in that the lung tissue of the animals showed elevated 5-HT7 expression.^19^ Additionally, the impact of 5-HT7 activation was investigated in alveolar basal epithelial human cells (A549) treated with LPS. Like in in vivo settings, LPS causes A549 cells to produce tumor necrosis factor α and more inducible nitric oxide synthase; this effect is countered by LP44, the 5-HT7 agonist. The upregulation of 5-HT7 expression in sepsis models may indicate a defensive function for the specific subtype.^19^ Furthermore, 5-HT7R might be part of a defense mechanism that comes from lung cells themselves, independent of immune or supportive cells.

Conversely, in an idiopathic pulmonary fibrosisin vivo model, 5-HT7R antagonistic activity demonstrated protective effects. Intratracheal bleomycin-induced increases in oxidative stress load, lung fluid volume, and inflammatory cytokine levels were all lessened by daily intraperitoneal injections of SB269970, 5-HT7R antagonist, in a rat model.^59^ The pathophysiology of the inflammatory state may determine the function of 5-HT7R in pulmonary inflammation.

### Cardiovascular Effects

Upon its discovery, serotonin was named by combining the terms “ser” for the serum it was extracted from and “tonin” to signify its ability to alter blood vessel tone in response to its effects. Where the pharmacological responses triggered by 5-HT7R are located within the cardiovascular system may have an impact on the intricate function of 5-HT7R. The main function of 5-HT7Rs on smooth muscle cells is to cause vasodilation in some arteries, including the cerebral, coronary, pulmonary, and aortic.^48^ On the other hand, 5-HT is known to be one of the strongest vasoconstrictor chemicals in the placental–umbilical circulation. According to these data, 5-HT7R expression was shown in normal placenta, and extremely increased expression of 5-HT7R was also shown in pre-eclamptic placentae in contrast to healthy.^22^ It has been proposed that the preeclamptic state’s elevated expression, which is linked to an augmented immune and inflammatory response, impaired endothelial function, and active coagulation, may be the cause of all of these pathologies.

Cardiovascular effects that are thought to be mediated by 5-HT7R include tachycardia in cats, prolonged hypotension in rats with vagosympathectomy, dilatation of the splanchnic vein, suppression of vagal-evoked bradycardia in rats linked to a drop in blood pressure in rats, and relaxing of the pulmonary arteries in weaned pigs due to 5-HT.^60-62^

Rats that were both awake and anesthetized showed decreased responses to baro-, chemo-, and cardiopulmonary reflex activation when the 5-HT7 antagonist SB269970 was administered intracisternal.^63,64^ The observed impact on all these reflexes implies that 5-HT7 in the brain stem could potentially aid in the processing of autonomic reactions triggered by the activation of cardiovascular reflexes. Additionally, in rats whose cardiovascular reflexes had previously been activated, the 5-HT7R antagonist SB-269970 raised blood pressure but not heart rate.^64^ This suggests a 5-HT7-mediated pathway that offers a normalizing input in response to problems brought on by the activation of the cardiovascular reflex. It has been suggested that 5-HT7R may play a complex role within the nucleus tractus solitarii, mediating these effects.^65^ In contrast, Cuesta et al^66^ demonstrated that 5-HT7R activation by an agonist AS-19 indirectly inhibits hypotension.

It is also shown that a high-fat diet increases 5-HT7R mRNA levels with endothelial damage, and isoproterenol addition exacerbates this increase in the heart. As agonist application observed that all pathological findings and inflammatory cytokines decreased in parallel with the decrease in 5-HT7R expression, it was reported that no pathological improvement was observed in the heart tissue with SB269970 application.^67^

The complex part of 5-HT7R in the cardiovascular system may be explained by the possibility that the location of 5-HT7-driven pharmacological responses in the cardiovascular system influences those responses.^16^

### Fibrosis and Carcinogenesis

It has been demonstrated that serotonin is a mitogenic agent for a variety of typical and malignant cells.^68^ As a result, 5-HTRs may be involved in the etiology of several cancer kinds. Serotonin 7 receptor is a possible target for therapy because it has recently been proposed that it may have a role in the physiopathology of some cancer types.

Seotonin is known to stimulate breast cancer cells’ oncogenic signaling pathways, including Akt/mTOR, MAPK, and JAK/STAT3/ERK.^69^ And the activation of 5-HT7 by 5-HT has been shown to cause a considerable reduction in proliferation in normal mammary epithelial cells; this effect is not present in some cell lines of breast cancer.^70,71^ Also, it has been concluded that higher tumor grades are associated with increased expression of 5-HT7R in some breast cancer types. The cell line of triple-negative breast cancer MDA-MB-23 cells’ reactivity to 5-HT was reduced when 5-HT7 was blocked, owing to distinct signaling pathways.^72,73^

Prostate carcinoma, benign prostate epithelium, and metastases have all been linked to serotonin pathways specific to malignancy. So, 5-HT7R has been investigated as a possible target for prostate cancer.^18^ It has been explored that, compared to healthy prostate tissue, malignant prostate tissue expresses more of the 5-HT7R mRNA. The SB-269970, an antagonist of 5-HT7Rs, has been demonstrated to reduce proliferation in PC-3 cells and to be associated with an apoptosis-inducing action. As a result, researchers suggested that inhibiting 5-HT7Rs could be a new therapeutic target for prostate cancer treatment.^18^

Additionally, there are many studies suggesting that 5-HT7R regulation may be useful as a therapeutic intervention for liver cancer. The pharmacological blockade of the 5-HT7R by the highly specific antagonist SB 269970 reversed the effect of serotonin’s dose-dependent activation of cAMP and PKA signaling in an in vitro study on rat hepatocytes. This finding may have implications for extra-hepatic tumor seeding in the liver.^23^

Another pathway for 5-HT, maybe through 5-HT7, stimulates the production of β-catenin and the growth of HCC. The 5-HT7 antagonist SB-258719 was found to be effective in reducing the 5-HT-induced rise in β-catenin levels and cell viability, and it also increased the expression of 5-HT7 in HCC cell lines. In an in vivomouse model of tumor xenografts, SB-258719 remarkably inhibited the growth of tumors and the proliferation of patient-derived primary HCC cultures.^74^ So these data show that 5-HT7 antagonists may be effective for the treatment of HCC.

Liver fibrosis is thought to be a frequent risk factor for HCC and is highly correlated with it. A model of developing cirrhosis created chemically showed decreased expression of 5-HT7, and liver tissue was shielded from oxidative damage by the 5-HT7 agonist LP-44, which markedly decreased the production of inflammatory cytokines and markers of liver damage.^21^ Contrary to the results seen in the previously mentioned HCC model, these findings suggest that 5-HT7 agonists may be useful in the management of persistent fibrosis and inflammation of the liver.

In addition, a study conducted considering the increase in serotonin in nasal polyps showed that 5-HT7 receptors are expressed in the nasal cavity, and 5-HT7 receptors are expressed at high levels in patients with nasal polyps.^75^

## Conclusion

It is important to maintain interest in this final member of the serotonin receptor family, given its wide distribution in various tissues and its immunomodulatory and oncogenic effects. Consequently, 5-HT7R modulating drugs appear to be a more practical treatment option, with selective enhancement or suppression depending on the pathological condition.
